# Particle Swarm Optimization-Based Approach for Optic Disc Segmentation

**DOI:** 10.3390/e24060796

**Published:** 2022-06-08

**Authors:** Junyan Yi, Ya Ran, Gang Yang

**Affiliations:** 1 Department of Computer Science and Technology, Beijing University of Civil Engineering and Architecture, Beijing 100044, China; yijunyan@bucea.edu.cn (J.Y.); 2108110019009@stu.bucea.edu.cn (Y.R.); 2 Information School, Renmin University of China, Beijing 100080, China

**Keywords:** particle swarm optimization, optic disc segmentation, subgroups, exploration area

## Abstract

Fundus segmentation is an important step in the diagnosis of ophthalmic diseases, especially glaucoma. A modified particle swarm optimization algorithm for optic disc segmentation is proposed, considering the fact that the current public fundus datasets do not have enough images and are unevenly distributed. The particle swarm optimization algorithm has been proved to be a good tool to deal with various extreme value problems, which requires little data and does not require pre-training. In this paper, the segmentation problem is converted to a set of extreme value problems. The scheme performs data preprocessing based on the features of the fundus map, reduces noise on the picture, and simplifies the search space for particles. The search space is divided into multiple sub-search spaces according to the number of subgroups, and the particles inside the subgroups search for the optimal solution in their respective sub-search spaces. The gradient values are used to calculate the fitness of particles and contours. The entire group is divided into some subgroups. Every particle flies in their exploration for the best solution. During the iteration, particles are not only influenced by local and global optimal solutions but also additionally attracted by particles between adjacent subgroups. By collaboration and information sharing, the particles are capable of obtaining accurate disc segmentation. This method has been tested with the Drishti-GS and RIM-ONE V3 dataset. Compared to several state-of-the-art methods, the proposed method substantially improves the optic disc segmentation results on the tested datasets, which demonstrates the superiority of the proposed work.

## 1. Introduction

According to statistics of the World Health Organization (WHO), glaucoma is the second leading cause of blindness in the world [1]. It is estimated that people with glaucoma will increase to 111.8 million in 2040 [2].

An early diagnosis of glaucoma is of great importance. Glaucoma is irreversible after blindness, which leads to structural modifications as the disease progresses [3]. Fortunately, glaucoma has a long lesions cycle, and the patient’s vision is slowly weakened. If detected and treated as early as possible, patients still can maximize the preservation of useful vision to maintain a normal life and work life [4]. Therefore, the early diagnosis and treatment of glaucoma are particularly necessary.

**Figure 1:**
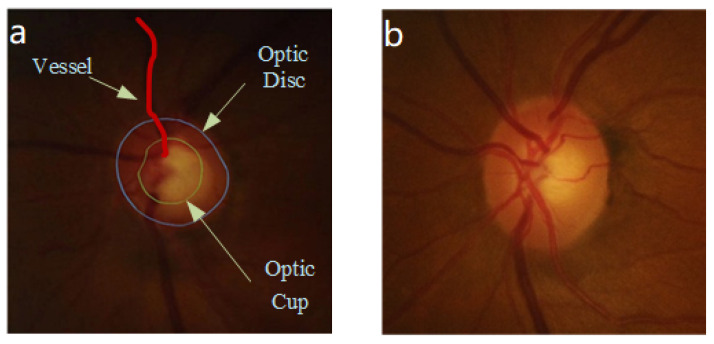
Retinal fundus of glaucoma and normal eyes. (**a**) Schematic diagram of the structure of fundus with glaucoma; (**b**) fundus of normal eyes.

In actual medical diagnosis, ophthalmologists usual use cup to disk ratio (CDR [5]) as one of the factors in diagnosing glaucoma. The optic disc (OD) in the digital fundus image is the area where blood vessels and optic nerve fibers enter the retina. In digital fundus images, OD appears as a bright oval area, and the optic cup (OC) is the brighter oval area in the center of OD [1]. When the ratio of optic cup to the optic disc that means the diameter of the cup divided by the diameter of the disc [6,7] is greater than 0.6, which is considered to indicate glaucoma [8]. Glaucoma and normal fundus images are shown in Figure 1. Figure 1a shows a fundus diagram with glaucoma, for which its CDR is much greater than 0.6, while Figure 1b is a fundus of the normal eye, which can be seen when the central bright area; that is the cup area, and it is much smaller than the optic disc area. Therefore, many scholars use computers as an aid for the early diagnosis of glaucoma. The effective division of the optic disc area on the fundus is of extraordinary significance to the diagnosis of glaucoma. In this paper, the segmentation of the OD region is the main research focus.

In recent years, different approach of optic disc segmentation such as machine learning and clustering have been proposed so far. Thus, a deep learning architecture called M-Net is proposed by Fu, H. [9], which uses U-shaped neural networks as the main frame and introduces polar coordinate transformations to solve the problem of OD segmentation in a multi-level label system. Al-Bander and Baidaa et al. [10] found a new method based on deep learning that separates OD in the fundus image by using a combination of a fully convolutional network and DenseNet. Although deep learning-based approaches obtain a satisfactory performances of optic disc segmentation, those approaches require a huge number of data for the time-consuming training required. On the basis of color similarity and image proximity, Achanta et al. [11] segmented the optic disc with the generation of superpixels using clustering. There is no doubt that superpixel methods do not consider color information, which may adversely impact performance.

There are many drawbacks when applying deep learning to the segmentation of optic discs. One of the main reason for this is that the glaucoma datasets are too small to meet the training requirements of deep learning. The datasets originate from hospitals, and to protect the privacy of the patient, the patient’s permission must be obtained [12]. Furthermore, the optic disc must hand labeled by experienced ophthalmologists, which undoubtedly increases the difficulty of obtaining datasets.

To overcome the drawback of the lack of glaucoma dataset, a new particle swarm optimization-based approach for optic disc segmentation SePSO is proposed in our work. The proposed algorithm does not require large amounts of data for preprocessing, which is a self-learning approach. In addition, the information of image color and optic disc shape is also taken into our consideration, which reduces the interference of relevant information on experimental results [13].

Furthermore, the subsequent sections are organized as follows: Section 2 describes details on the PSO approach that we modified for the segmentation of optic disc. Section 3 provides some results with datasets, evaluation parameters used for our experimental study, and the discussion. Moreover, the conclusion is highlighted in Section 4.

## 2. Materials and Methods

This section described the Particle Swarm Optimization algorithm that we modified in this paper to segment the optic disc in retinal images. As described by Shi and Eberhart [14], PSO is a multi-agent system, and the system is initialized with a population of random solutions. For each potential solution, called particle, all particles fly in the exploration area for the global optimum. Each particle tracks its coordinates in exploration area. The coordinates of each particle tracks are associated with the best solution [15] (fitness) achieved by itself so far, which is called PP, and the entire group’s best solution, which is called GP. The particle can be described as $Par_{i,j}=\left(X_{i,j},V_{i,j},PP_{i}\right)$, where $X_{i,j}$ is the current position of particle, and $V_{i,j}$ is the current velocity of particle. Each particle moves toward PP and GP at every iteration.

The basic idea of PSO is to find the optimal solution through collaboration and information sharing between individuals in the animal populations. Usually, the PSO algorithm can be used to optimize continuous nonlinear functions and to solve some discrete problems.

In this paper, the optic disc segmentation problem is converted into a series of the extreme value problem. Based on a large number of experiments, we found that it is not sufficient to solve the optic disc segmentation problem by original PSO. To solve this problem, we propose an improved algorithm based on Particle Swarm Optimization, called SePSO. The goal of SePSO is to find a set of best solutions for the particles. Moreover, the closed shape formed by these particles is the boundary of the predicted optic disc. The components of the SePSO algorithm we modified are described in subsequent sections.

### 2.1. Subgroups and Edges

In this paper, the PSO approach has been used in segmenting OD in a fundus image. In order to achieve this goal, the concept of subgroups are drawn into PSO. The entire particle group is divided into a certain number of subgroups pop. There are *N* subgroups in the original group, where the subgroups consist of the same number of particles $n_{p}$. Particles in every subgroups are marked with numbers from 1 to $n_{p}$. For a clear expression, $Par_{i,j}$ represents a particle with a label of No.j in the *i*th subgroups. Each subgroup $pop_{i}$, which searches within its respective regions (details in Section 2.2.1), contains some particles $Par_{i,j}$. The subgroups pop can be described as Equation (1):

(1) $$pop_{i}=\left\{Par_{i,1},Par_{i,2},\ldots ,Par_{i,n_{p}}\right\}$$

where

- $pop_{i}$ is the *i*th subgroup;
- The parameter i=1,2,…,N;
- $n_{p}$ is the number of particles belonging to every single subgroup;
- $Par_{i,j}$ is the particle marked as No.j in subgroup $pop_{i}$.

The closed curve, which consists of all the No.j particles in each subgroups connected, is called $Edge_{j}$ in this paper. The number of potential boundary is the same as the number of particles in each subgroup, which is $n_{p}$. In addition, Edge is the potential solution of optic disc boundary. Edge can be defined as Equation (2):

(2) $$Edge_{j}=\left\{X_{1,j},X_{2,j},\ldots ,X_{N,j}\right\}$$

where

- $Edge_{j}$ is the *j*th potential OD boundary;
- The parameter $j=1,2,\ldots ,n_{p}$;
- *N* is the number of subgroups;
- $X_{N,j}$ is the position of No.j particle in subgroup $pop_{N}$.

As the search progresses, most particles in each subgroup converge to the optimal solution and the Edge with best solution (fitness, details in Section 2.3) makes up the final predicted boundary.

### 2.2. Exploration Area and Initiation

#### 2.2.1. Exploration Area

**Figure 2:**
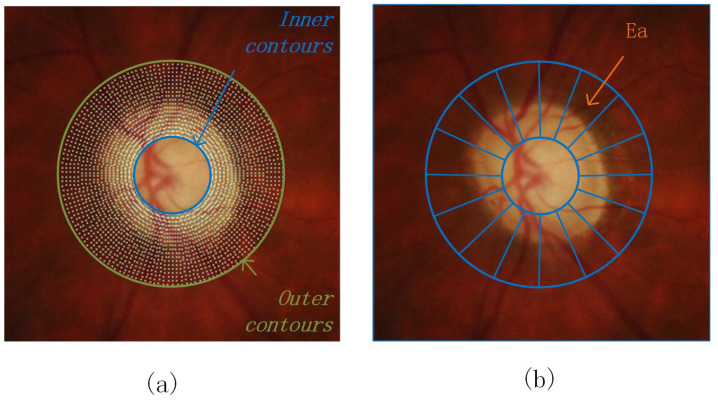
Diagram of the exploration area. (**a**) The blue and yellow lines, respectively, represent the internal and external contours of exploration area, and the besieged point is the radial points map EA. (**b**) Ea is the exploration area of each subgroups.

Using the SePSO algorithm for OD segmentation means that each particle searches in the two-dimensional space for a set of Edge. Obviously, the entire two-dimensional space in original image is too broad for all the particles to search. It is advisable to optimize the exploration area first. To simplify the search processing, reduce the computation time and increase the accuracy of OD segmentation; we pre-process the original images in three steps. Figure 2 shows the diagram of exploration area.

Firstly, the ROI images prepared for OD segmentation are initially resized to 256×256 pixels by using the interpolation of a Lanczos interpolation [16] over an 8 × 8 pixel neighborhood. On one hand, making the size of images homogeneous is helpful for the subsequent calculations and evaluations. On the other hand, the exploration area can be compressed as small as possible on the premise of preserving features.

Secondly, according to prior knowledge and the circle nature of the optic disc, we set the entire exploration area as an annular area. Considering the center of the disc as the center $P_{c}$ of the entire exploration area, the outer $E_{outer}$ and inner $E_{inner}$ exploration contours are, respectively, defined by the circle of center $P_{c}$ and radius $R_{max}$ and $R_{min}$, which are defined as Equation (3).

(3) $$\begin{array}{c} E_{outer}=\left\{\left(x,y\right)|x^{2}+y^{2}=R_{max}^{2}\right\}\\ E_{inter}=\left\{\left(x,y\right)|x^{2}+y^{2}=R_{min}^{2}\right\} \end{array}$$

In addition, a polar transform [17] is applied to OD segmentation. The entire exploration area consists of a series of radial points, which is defined as a radial points map EA. The radial points map is a two-dimensional polar coordinate system, and the points represent where particles are likely to explore. The radial points of this map are described by distance and angle, where the polar coordinate of $P_{c}$ is (0,0). Rays that proceed horizontally to the right from $P_{c}$ are called polar axes [18]. The distance between the search points and center point $P_{c}$ is called radius $r\in [R_{min},R_{max}]$. The angle is calculated by gradually adding an increment of $\delta_{\theta }$. Therefore, the location of pixels on the map is described with a radius and angle by Equation (4):

(4) $$\begin{array}{c} \forall i\in Z\cap \left[0,\frac{R_{max}-R_{min}}{\delta_{r}}\right],j\in Z\cap \left[0,\frac{2\pi }{\delta_{\theta }}\right]\\ \begin{array}{cc} P_{i,j} & =(r_{i,j},\theta_{i,j})\\ \; & =(R_{min}+\delta_{r}\times i,\delta_{\theta }\times j) \end{array} \end{array}$$

where

- $P_{i,j}$ is the particle in the exploration map;
- $r_{i,j}$ is the distance between $P_{i,j}$ and $P_{c}$;
- $\theta_{i,j}$ is the angle of $P_{i,j}$.

Therefore, the Cartesian coordinates of pixel $P_{i,j}$ is described with radius and angle by Equation (5).

(5) $$\begin{array}{c} \forall i\in Z\cap \left[0,\frac{R_{max}-R_{min}}{\delta_{r}}\right],j\in Z\cap \left[0,\frac{2\pi }{\delta_{\theta }}\right]\\ \begin{array}{cc} P_{i,j} & =(x_{i,j},y_{i,j})\\ \; & =(x_{c}+r_{i,j}\times \cos\theta_{i,j},y_{c}+r_{i,j}\times \sin\theta_{i,j}) \end{array} \end{array}$$

where

- $x_{i,j}$ and $y_{i,j}$ is the horizontal and vertical ordinate of $P_{i,j}$;
- $x_{c}$ and $y_{c}$ is the horizontal and vertical ordinate of $P_{c}$.

The entire exploration area EA consists of a set of pixels, which can be described as Equation (6).

(6) $$EA=\{P_{i,j}:\forall \left(i,j\right)\in Z\cap \left[0,\frac{R_{max}-R_{min}}{\delta_{r}}\right],Z\cap \left[0,\frac{2\pi }{\delta_{\theta }}\right]\}$$

(7) EA=(V,E)

Similarly to the relationship between POP and pop, the exploration area of subgroups SE is not only defined as part of the entire exploration area or any two regions that do not intersect. Equations (8) and (9) are the properties and definition of $SE_{i}$:

(8) $$\begin{array}{c} \forall \;i\in Z\cap [1,N]\\ EA=\overset{N}{\underset{i=1}{⋃}}Ea_{i}\\ \overset{N}{\underset{i=1}{⋂}}Ea_{i}=\emptyset \end{array}$$

(9) $$SE_{i}=\left\{P_{i,j}:\forall \left(i,j\right)\in Z\cap \left[0,\frac{R_{max}-R_{min}}{\delta_{r}}\right],Z\cap \left[\frac{2\pi }{\delta_{\theta }}\times \left(i-1\right),\frac{2\pi }{\delta_{\theta }}\times i\right]\right\}$$

where $SE_{i}$ is the exploration area of subgroup $pop_{i}$.

#### 2.2.2. Initiation

The shape of the optic disc varies from circle to ellipse, and usually it is an irregular approximation of the ellipse. If too few points are used for fitting, it is difficult to express the true disc’s boundary. The more points are used for fitting, the closer the fitted shape is to the real boundary. However, too many points will slow down the calculation and increase time complexity. We need to find the proper number of points to balance speed and accuracy. In this paper, the number of points on the boundary Edge is equal to the number of subgroups *N*. The influence of the parameter *N* will be shown in subsequent experiments.

In PSO, the particle number $n_{p}$ of each subgroup usually is between 20 and 40. The more particles there are, the wider the search space is. It is easy for SePSO to find the global optimal solution with more particles; however, computational consumption will become higher.

In this paper, the particle number $n_{p}$ is set as $n_{p}=30$ based on a large number of experiments.

Each particle is assigned a randomized location at the beginning. Considering of the circle nature of OD, the distances from particles on the predicted boundary $Edge_{j}$ to $P_{c}$ should be equal approximately for each *j*. Therefore, the initial location of each particle is set using the following rules:

- For all particles $Par_{i,j}$ belonging to the same $Edge_{j}$ having the same radius *r*, *r* is the distance between $Par_{i,j}$ and center $P_{c}$.
- The radius *r* of particles $Par_{i,j}$ on $Edge_{j}$ is randomly assigned from $R_{min}$ to $R_{max}$. Based on experiments, $R_{min}$ is set as 30% of the image width, and $R_{min}$ is the 70% of the image width.

### 2.3. Fitness Function

**Figure 3:**
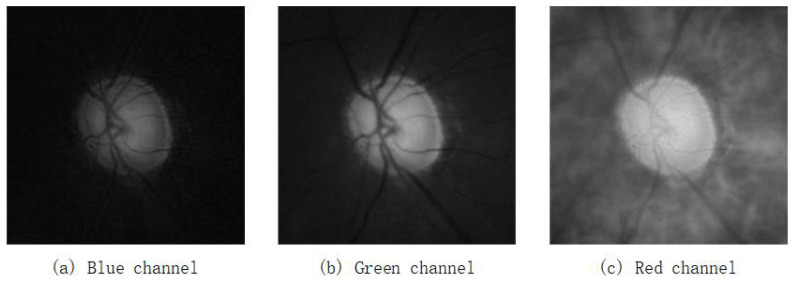
The different channel of ROI image in Rim-ONE.

**Figure 4:**
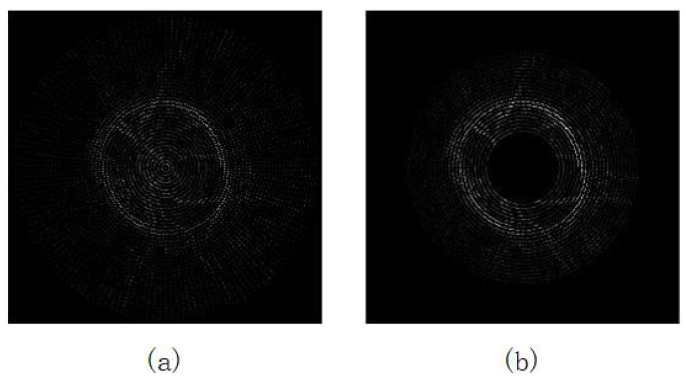
Comparison of the gradient between different methods. (**a**) The gradient in original exploration area. (**b**) The gradient in the modified exploration area EA.

When particles fly in the subgroup’s exploration area Ea, an indicator should be introduced to evaluate the locations, and a fitness function is used as the indicator in this paper. In the task of image segmentation, considering the differences between the OD region and the surrounding space, the red channel of the original RGB image is separated to calculated the gradient of every radial points in the exploration area [19]. The differences between the images of blue, green, and red channels are showed in Figure 3. The gradient is regarded as the fitness value of points. Figure 4 shows the different gradient between original exploration and modified exploration. The fitness value of each point is calculated as Equation (10).

We have the following equation:

(10) $$value_{P}=\left\{\begin{array}{c} grad_{P},when\;P\in EA\\ 0,when\;P\notin EA \end{array}\right.$$

where

- $value_{P}$ is the fitness value of point *P*;
- $grad_{P}$ is the gradient [20] of point *P*.

In addition, the potential contours of OD, which is described as $Edge_{j}$, consists of the *j*th particle in every subgroup $pop_{i}$. The higher the fitness of a point, the more likely it is that the point is part of the boundary. The higher the fitness value is, the higher the possibility that it is part of Edge. The fitness of $Edge_{j}$ can be calculated with Equation (11):

(11) $$value_{Edge_{j}}=\sum_{i=1}^{N}value_{i,j}$$

where $value_{i,j}$ is the fitness of the *j*th particle in subgroup $pop_{i}$.

### 2.4. Position Update

In the traditional PSO, each particle changes the location toward the particle’s best location PP and the global best location GP at each step. There are three control parameters, the inertia weight ω, the cognitive $c_{p}$, and social $c_{g}$ acceleration, that play an important part in the exploration and exploitation capability of PSO. The velocity of every particle can be calculated as Equation (12):

(12) $$\begin{array}{c} V_{i,j}\left(t+1\right)=\omega \times V_{i,j}\left(t\right)+c_{p}\times rand\times \left(PP_{i,j}\left(t\right)-X_{i,j}\left(t\right)\right)+c_{g}\times rand\times \left(GP_{i}\left(t\right)-X_{i,j}\left(t\right)\right) \end{array}$$

where

- $V_{i,j}$ is the velocity of the *j*th particle in subgroup $pop_{i}$;
- ω is the inertia weight;
- rand∈[0,1], which is a random number;
- $c_{p}$ and $c_{g}$ are named as acceleration coefficients. Usually, $c_{p}+c_{g}=4$;
- $PP_{i,j}$ is the best location of $Par_{i,j}$ has flied;
- $GP_{i}$ is the best location of the entire subgroup $pop_{i}$.

However, after a series of experiments (see Section 3.3.1), the fitness of the vessel boundary in the fundus image may be greater than the gradient of optic disc boundary, which is possible to interfere with the optimization results of the particles. Therefore, we take the attraction between particles in adjacent subgroups into account in order to solve this problem.

The modified velocity is calculated as Equation (13).

(13) $$\begin{array}{c} V_{i,j}\left(t+1\right)=\omega \times V_{i,j}\left(t\right)+c_{p}\times rand\times \left(PP_{i,j}\left(t\right)-X_{i,j}\left(t\right)\right)+c_{g}\times rand\times \left(GP_{i}\left(t\right)-X_{i,j}\left(t\right)\right)\\ +c_{a}\times rand\times \left(X_{i+1,j}\left(t\right)-X_{i,j}\left(t\right)\right)+c_{a}\times rand\times \left(X_{i-1,j}\left(t\right)-X_{i,j}\left(t\right)\right) \end{array}$$

As the system iterates, the individual agents, which are based on the interaction of the subgroup’s public search and the particle’s search, are drawn toward a global optimum. The position vector of each particle is updated as Equation (14).

(14) $$X_{i,j}\left(t+1\right)=X_{i,j}\left(t\right)+V_{i,j}\left(t+1\right)$$

PP and GP are also updated as iteration proceeds. The updated rules are described by Equations (15) and (16).

(15) $$PP_{i,j}\left(t+1\right)=\left\{\begin{array}{c} PP_{i,j}\left(t\right),\;if\;value_{PP_{i,j}}\left(t\right)>value_{P_{i,j}}\left(t+1\right)\\ P_{i,j}\left(t+1\right),\;if\;value_{P_{i,j}}\left(t+1\right)\leq value_{PP_{i,j}}\left(t\right) \end{array}\right.$$

(16) $$GP_{i}\left(t+1\right)=\left\{\begin{array}{c} GP_{i}\left(t\right),\;if\;value_{GP_{i}}\left(t+1\right)>value_{PP_{i,j}}\left(t\right):\forall j\in Z\cap \left[1,n_{p}\right]\\ PP_{i,j}\left(t+1\right),\;if\;value_{GP_{i}}\left(t+1\right)\leq value_{PP_{i,j}}\left(t\right):\exists j\in Z\cap \left[1,n_{p}\right] \end{array}\right.$$

### 2.5. Algorithm

SePSO evaluates the quality of particle positions by using the radial gradient value as the fitness value. On fundus diagrams, the location with high radial gradient values include the optic disc boundary, the optic cup boundary, and the vascular region. In order to segment the clear boundary of the optic disc and reduce the interference of blood vessels and optic cup located in the central area of the optic disc, the exploration area of the entire population is confined to a relatively narrow circular area. This exploration area can not only meet the exploration needs of particles but also avoid particles falling into local optimization as much as possible. In addition, the application of active shape models transforms image segmentation tasks into a set of extreme value problems that are easy to solve by PSO. To implement this mechanism, we divide the entire particle swarm into multiple subgroups pop, and at the same time, we divide the exploration space into multiple subspaces accordingly; each subgroup solves an extreme value problem, each particle on the subgroup corresponds to a feature point on the shape model, and all subgroups form a multi-group solution of the shape model. The quality of the shape model $Edge_{j}$ is determined by the fitness of all particles that make up the model at their current positions.

In the search for an optimal solution, each particle is represented by a triplet ($X_{i,j}$, $V_{i,j}$, and $PP_{i,j}$), and shape model $Edge_{j}$ is defined by the position of a set of particles. The boundary of the disc is an approximate ellipse, and the position of adjacent particles on the shape model has some guiding significance for the particle. To take full advantage of this property, particles are attracted to the local optimal solution and the global optimal solution. Moreover, the movements of the particles are also affected by the attractions between adjacent particles. Shape model $Edge_{j}$ deforms as the iterative process progresses, ultimately finding the optimal solution.

The main flow of the proposed Algorithm 1 is shown in the pseudo-code.
**Algorithm 1:** Pseudo-code for the main particle management algorithm.  **input**  : A map of exploration area  **output**: A set of points
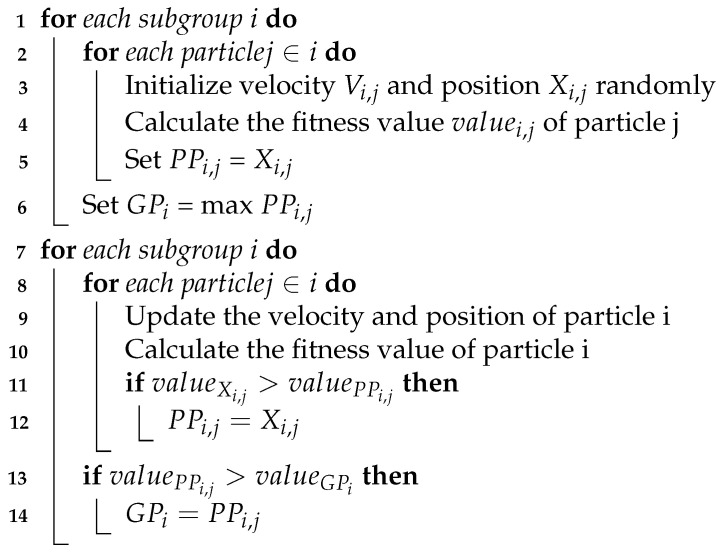


## 3. Results and Discussion

This section describes the hardware facilities, software, and dataset used in the experiment and, furthermore, includes the evaluation and performance metrics of the proposed approach.

### 3.1. Software and Datasets

All experimental results are tested on PyCharm 2021 free community version with Python 3.8.7, AMD “Zen 3” Core Architecture with 5600X CPU and 64-bit Operating System. In addition, the fundus images used for our experimentation belongs to public datasets provided below.

The Drishti-GS is public access and available for free [21]. The dataset consists of 101 images, which is divided into 50 testing and 51 training images. The dataset is generated from Aravind eye hospital. There are four experts to segment the OD region manually, and all OD regions are fused together to generate the soft map. In this paper, we regard the region with 75% support rate as the true ground of OD.

Up to date, there are three releases of Rim-ONE datasets. The first one is segmented by five experts manually, which consists of 169 images. The second one consisting of 455 images segmented by one expert is classified into glaucoma (including the glaucoma suspicious images) and normal. The most recent one, which is used for our experimentation, consists of 159 images [22], which is collected at the Hospital Universitario de Canarias and divided into 85 images of healthy subjects, 39 confirmed glaucoma individuals, and 35 that are suspicious subjects.

### 3.2. Performance Metrics

The performance of the proposed method for segmenting OD when compared with the ground truth is evaluated using many evaluation metrics that can be defined according to four parameters: $T_{N}$, $F_{N}$,$T_{P}$, and $F_{P}$.

True Negative $T_{N}$ is the region segmented as OD that proved to be not OD and is defined as Equation (17):

(17) $$T_{N}=\left\{\begin{array}{c} 1-\frac{SA-OA}{BA_{O}A},\;if\;SA>OA\;\\ 1,otherwise \end{array}\right.$$

where

- SA is the optic disc region that is segmented;
- OA is the region that belongs to ground truth;
- SA is the region that belongs to the background.

False Negative $F_{N}$, which is the region segmented as not OD that proved to be OD, is defined in Equation (18).

(18) $$F_{N}=\left\{\begin{array}{c} 0,\;if\;SA\geq OA\;\\ \frac{OA-SA}{OA},otherwise \end{array}\right.$$

True positive $T_{P}$, which is the region segmented as OD that proved to be OD, is defined in Equation (19).

(19) $$T_{P}=\left\{\begin{array}{c} 1,\;if\;SA\geq OA\;\\ 1-\frac{OA-SA}{OA},otherwise \end{array}\right.$$

False Positive $F_{P}$, which is the region segmented as OD that proved to be not OD, is defined in Equation (20).

(20) $$F_{P}=\left\{\begin{array}{c} \frac{SA-OA}{BS-OA},\;if\;SA>OA\;\\ 0,otherwise \end{array}\right.$$

In this paper, the evaluation metrics such as Dice similarly, overlapping error, and accuracy are used to identify the level of accuracy of OD segmentation, which can be defined as Equations (21)–(23).

*Accuracy:* It is the measure of correctness or preciseness with respect to some standard [23]. It is calculated as provided in Equation (21).

(21) $$Acc=\frac{T_{N}+T_{P}}{T_{N}+F_{N}+F_{P}+T_{P}}$$

*Dice similarity:* It is a metric that is used to calculate the similarity between two samples that may be images or any other data. It is calculated as provided in Equation (22).

(22) $$Dice=\frac{2\times T_{P}}{2\times T_{P}+F_{P}+F_{N}}$$

*Overlapping error:* Overlapping error *E* is the one of the important indicators of image segmentation. In this paper, it is calculated using the detected boundary and the ground truth by Equation (23).

(23) $$E=1-\frac{T_{P}}{T_{P}+F_{P}+F_{N}}$$

### 3.3. Performance of Optic Disc Segmentation

#### 3.3.1. Parameter Influence

In order to obtain the best results with proposed method, there are some important parameters that should be adjusted. These parameters are as follows: the number of subgroups *N*, which affect the Edge in Section 2.1; ω, $c_{p}$, $c_{g}$, and $c_{a}$, which affect the position update as described in Section 2.4; and the number of iterations.

Based on a large number of experiments on dataset, the proposed method produces the best solutions with the following parameter settings: N=50, ω=0.8, $c_{p}=c_{g}=1.7$, $c_{a}=0.3$, and number of iteration = 100 (see Table 1). The values of $T_{P}$, $F_{P}$, $T_{N}$, $F_{N}$, Acc, Dice, and *E* produced by the proposed approach for optic disc segmentation are observed to be 84.88%, 0.73%, 99.27%, 15.12%, 92.28%, 90.35%, and 17.49% in the Drishti-GS dataset.

We have varied each parameter individually to observe its influence. Some of these parameters mainly affect the quality of the solutions, while some have more impact on the computation time needed to obtain the solutions.

**Figure 5:**
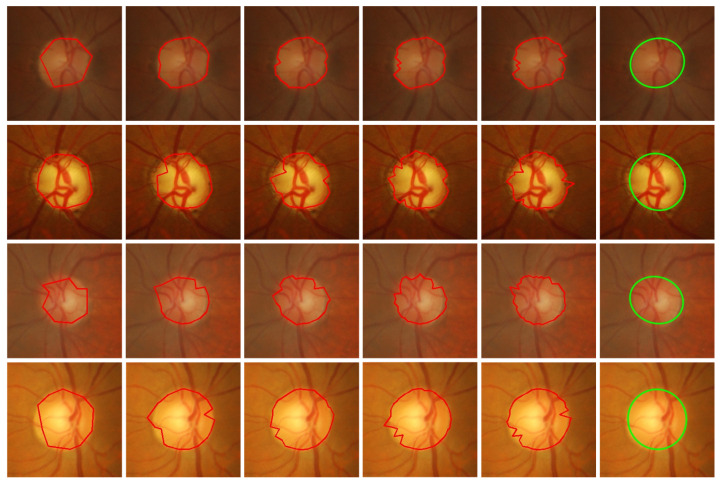
Optic disc segmentation in Drishti-GS with different numbers of subgroups and the shape of the ellipse’s fit. From left to right, the number of subgroups is 10, 20, 30, 40, and 50. The last column is the result of the interpolation fitting based on column 5.

*The number of subgroups N:* This parameter affects the number of points that make up the boundary. The higher the value *N*, the closer the fitted shape is to the true boundary. However, too many subgroups increase the calculation time’s complexity. There are some experiments that show the impact of this parameter on accuracy and calculation time. Table 2 provides *E*, Acc and the calculation time for a different number of subgroups in Drishti-GS. Figure 5 shows the optic disc segmentation in Drishti-GS with different numbers of subgroups. It can be seen clearly from Table 2 and Figure 5 that, when the number of subgroups equals to 50, good results can be achieved. In fact, if the number of subgroups is set to a higher value, better solutions can be obtained.

*$c_{p}$, $c_{g}$, and $c_{a}$:* These parameters affect the velocity of particles. $c_{p}$ and $c_{g}$ are used to ensure that particles move toward the best global location. Usually, $c_{p}=c_{g}=2$ in the traditional PSO algorithm. Therefore, the experiments start by setting these two parameters $c_{p}$ and $c_{g}$ to 2. The predict boundaries are not sufficient to fit ground truth boundary. The reason is that it is difficult to converge to optimal solutions of all the subgroups. Therefore, we introduce the attraction between adjacent particles by $c_{a}$. Table 3 provides the *E*, Acc and Dice for different parameter settings in Drishti-GS. It can be seen that the algorithm can performs optimally when $c_{p}=c_{g}=1.7$ and $c_{a}=0.3$.

*The inertia weight ω:* To obtain a better accuracy of OD segmentation with proposed method, an inertia weight strategy is applied to our experiments. Inertia weight ω is an important parameter in PSO, which significantly affects the convergence and exploration in PSO processes. Since the inception of inertia weight in PSO, a large number of variations of inertia weight strategy have been proposed [24]. In this paper, we compared the constant inertia weight, linear descending inertia weight, random inertia weight, and chaotic random inertia weight based on Drishti-GS dataset. Table 4 shows the different strategies of inertia weight. The parameter settings of all those experiments are N=50, $c_{p}=c_{g}=1.7$, $c_{a}=0.3$, and numberofiteration=100.

Table 5 provides E, Acc, and Dice for inertia weights with different strategies. It can be observed that the algorithm can perform optimally with the chaotic random inertia weight, which is 0.75% higher in Acc than the linear descending inertia weight, 0.17% higher in Dice, and 0.28% lower in E than the constant inertia weight.

#### 3.3.2. Comparison with Other Methods

A recent comparison of optic disc segmentation methods was presented in [29] using Rim-ONE and Drishti-GS datasets. Those datasets contain disc segmentations of 169 and 101 images, and they are publicly available and are free. Table 6 shows the evaluation results for disc segmentation using different methods in Rim-ONE and Drishti-GS datasets. Moreover, the information in Table 6 is quoted from the article of N. Thakur et al. [29] in 2019.

As we can see, the proposed SePSO algorithm performs better in the Drishit-GS dataset than the best method (region growing) in the performance parameter Acc. The algorithm performs better on the Rim-ONE dataset and 2.59% higher than the region-based growth algorithm that is optimal among the four algorithms and 1.81% higher on Drishti-GS dataset. To further show performance and increase the comparability of the proposed method, additional evaluation indicators are used, as shown in Table 7.

## 4. Conclusions

In this paper, we propose an improved method based on particle swarm optimization, called SePSO, for optic disc segmentation in retinal fundus images. In the disc segmentation task, the deformation of the segmented contour is completed by changing the position of the particles, and the optimal contour is solved by iterative update. In addition, the constraint equations for particle position and velocity are optimized. The particles are additionally attracted by adjacent subgroups in addition to the attraction of local optimal solutions and global optimal solutions during flight. Based on Drishti-GS, we test the influence of different parameter of the proposed method on the segmentation effect.

Particles in subgroups can learn optimal solution information between adjacent populations in the process of optimization, which greatly enhances the anti-interference ability of particles. The proposed method has been tested using Rim-ONE and Drishti-GS datasets and compared to other state-of-the-art methods, such as methods based on superpixels, contours, thresholds, and region growth method. Experimental results show that the algorithm performs better on both datasets than the other four algorithms, which confirms its effectiveness and superiority.

In future studies, we aim to conduct the proposed SePSO approach for solving optic cup segmentation. The simultaneous division of OC and OD has great clinical medical value, and the segmentation of the OC will become the next research content of our work.

## Figures and Tables

**Table 1:** Performance metrics for the best solution of optic disc segmentation produced by the proposed method for Rim-ONE and Drishti-GS datasets.

Dataset	$T_{P}$	$F_{P}$	$T_{N}$	$F_{N}$	Acc (%)	Dice (%)	E (%)
Drishti-GS	0.85	0.01	0.99	0.15	92.28	90.20	17.71
Rim-ONE	0.90	0.02	0.98	0.10	94.31	89.85	17.93

**Table 2:** *E*, Acc, and calculation time for different numbers of iteration in Drishti-GS.

Number of Subgroups	10	20	30	40	50
E (%)	23.14	18.49	17.76	17.73	17.54
Acc (%)	86.81	91.57	92.05	92.19	92.28
Calculation time (s)	2.60	8.20	17.35	28.40	45.04

**Table 3:** *E*, Acc, and Dice for different combinations of $c_{p}$, $c_{g}$, and $c_{a}$.

	$T_{P}$	$F_{P}$	$T_{N}$	$F_{N}$	Acc (%)	Dice (%)	E (%)
$c_{p}$ = $c_{g}$ = 2, $c_{a}$ = 0	0.86	0.01	0.99	0.14	92.28	90.07	17.96
$c_{p}$ = $c_{g}$ = 1.7, $c_{a}$ = 0.3	0.85	0.01	0.99	0.15	92.28	90.35	17.49
$c_{p}$ = $c_{g}$ = 1.5, $c_{a}$ = 0.5	0.85	0.01	0.99	0.15	92.28	90.32	17.54

**Table 4:** Inertia weight with different strategies.

Name of Inertia Weight	Formula	Reference
Constant inertia weight	ω=c	[25]
Linear descending inertia weight	$\omega_{k}=\omega_{max}-\frac{\omega_{max}-\omega_{min}}{item_{max}}\times k$	[26]
Random inertia weight	$\frac{1+rand}{2}$	[27]
Chaotic random inertia weight	z=rand, z=4×z×(1−z), $\omega =\frac{z+rand}{2}$	[28]

**Table 5:** *E*, *Acc*, and *Dice* for inertia weight with different strategies.

	Acc (%)	Dice (%)	E (%)
Constant inertia weight	92.18	90.08	17.92
Linear descending inertia weight	92.17	90.14	17.82
Random inertia weight	92.18	90.14	17.81
Chaotic random inertia weight	92.92	90.25	17.64

**Table 6:** Performance measures of approaches applied for optic disc segmentation.

Segmentation Approach	Dataset	$T_{P}$	$F_{P}$	$T_{N}$	$F_{N}$	Acc (%)
Superpixel classification [30]	Drishti-GS	0.81	0.13	0.86	0.18	88.01
	Rim-ONE	0.81	0	1.00	0.18	88.67
Contour based [31]	Drishti-GS	0.81	0.13	0.86	0.18	88.98
	Rim-ONE	0.82	0	1.00	0.17	89.78
Thresholding [32]	Drishti-GS	0.81	0.12	0.87	0.18	90.00
	Rim-ONE	0.82	0	1.00	0.17	90.45
Region growing [33]	Drishti-GS	0.82	0.12	0.87	0.17	90.05
	Rim-ONE	0.82	0	1.00	0.17	90.72
Proposed approach	Drishti-GS	0.85	0.01	0.99	0.15	92.28
	Rim-ONE	0.90	0.01	0.99	0.10	94.31

**Table 7:** The result of OD segmentation with SePSO in Drishti-GS.

E≤0.1	E≤0.2	E≤0.3	E≤0.4	E≤0.5	*E*	Acc	Dice
5.94%	71.29%	99.01%	100.00%	100.00%	17.64%	92.2%	90.25%

## Data Availability

MDPI Research Data Policies at http://cvit.iiit.ac.in/projects/mip/drishti-gs/mip-dataset2/Home.php and http://medimrg.webs.ull.es/research/retinal-imaging/rim-one/ (accessed on 7 May 2022).
